# Association of neuroticism with incident dementia and cognitive function: 26-year follow-up of EPIC-Norfolk study

**DOI:** 10.1093/ageing/afaf339

**Published:** 2025-11-23

**Authors:** Yaqing Gao, Robert N Luben, Shabina Hayat, Carol Brayne, Najaf Amin, Cornelia van Duijn, David Hunter, Thomas Littlejohns

**Affiliations:** Nuffield Department of Population Health, University of Oxford, Oxford, Oxfordshire, UK; Department of Public Health and Primary Care, University of Cambridge, Cambridge, Cambridgeshire, UK; Department of Behavioural Science and Health, University College London, London, UK; Cambridge Public Health and Department of Psychiatry, University of Cambridge, Cambridge, Cambridgeshire, UK; Nuffield Department of Population Health, University of Oxford, Oxford, Oxfordshire, UK; Nuffield Department of Population Health, University of Oxford, Oxford, Oxfordshire, UK; Nuffield Department of Population Health, University of Oxford, Oxford, Oxfordshire, UK; Department of Epidemiology, Harvard TH Chan School of Public Health, Boston, MA, USA; Nuffield Department of Population Health, University of Oxford, Oxford, Oxfordshire, UK

**Keywords:** neuroticism, dementia, cognitive function, life-course epidemiology, personality, older people

## Abstract

**Background:**

Neuroticism is a stable personality trait associated with increased vulnerability to mental and physical disorders. This study examined whether neuroticism is associated with the risk of dementia, particularly across the adult life course and over long-term follow-up.

**Methods:**

We analysed data from 19,678 dementia-free participants (mean [standard deviation, SD] age, 60.8 [9.3] years) who had neuroticism assessed between 1996 and 2000. Incident dementia was identified via linked hospital inpatient, mental health and mortality records through 2022. Cox proportional hazards models estimated hazard ratios (HRs) per 1-SD increase in neuroticism. Secondary analyses examined interactions, mediation and associations with cognitive performance on eight tests.

**Results:**

Over a median follow-up of 22.7 years, 2488 participants developed dementia. Neuroticism was associated with increased dementia risk in a dose–response manner (HR per 1-SD: 1.14; 95% CI: 1.10–1.19). The association persisted even after ≥20 years of follow-up (1.09 [1.01–1.17]) and across baseline ages 41–60 (1.16 [1.04–1.30]), 60–70 (1.11 [1.04–1.18]) and 70–81 years (1.14 [1.07–1.22]). Associations were stronger among *APOE* ε4 carriers and heavy drinkers, and may be partly explained by depression, hypertension and ischaemic heart disease. Higher neuroticism was linked to poorer cognitive function, particularly episodic memory and to impairment across more cognitive domains.

**Discussion:**

Neuroticism was associated with increased long-term dementia risk and poorer cognitive performance across mid- and later life, supporting its role in disease development rather than merely reflecting prodromal symptoms. Addressing vascular and mental health in high-neuroticism individuals may offer opportunities for dementia risk reduction.

## Key Points

- Higher neuroticism is linked to greater dementia risk across mid- to later life.
- Associations persisted when neuroticism was measured more than 20 years before dementia onset.
- Stronger associations were observed in *APOE* ε4 carriers and individuals with high alcohol intake.
- Depression, hypertension and ischaemic heart disease partly mediated the neuroticism–dementia link.
- Higher neuroticism is linked to poorer cognition across domains, especially episodic memory.

## Introduction

Neuroticism, a personality trait characterised by a tendency towards negative emotions, is a well-established risk factor depression, anxiety and cardiovascular diseases [1–3], and is implicated in dementia risk [4–7]. A meta-analysis of 12 longitudinal studies (*N* = 33 054) reported a 24% higher dementia risk per standard deviation (SD) increase in neuroticism, based largely on minimally adjusted models [4]. Two subsequent large UK Biobank (UKB) studies (both *N* > 100 000) reported 18% and 11% higher risk over mean follow-ups of 8.9 and 13.5 years [5, 6], respectively, after full adjustment for socioeconomic and lifestyle factors, with evidence of independence from genetic risk and partial mediation by mental and vascular conditions [6].

Despite growing evidence, key gaps remain. First, most prior studies had mean follow-up durations shorter than 10 years. Given the long preclinical phase of dementia [8], associations observed over shorter periods may reflect reverse causation, with personality change as an early manifestation of disease [9]. Notably, a large meta-analysis reported that neuroticism increases modestly during the transition to and progression of mild cognitive impairment and dementia [10]. To disentangle directionality, prospective studies with longer follow-up are needed. If neuroticism is primarily a prodromal marker, associations should attenuate over extended follow-up.

Second, no studies have examined whether associations vary by life stage. This is critical not only for disentangling directionality—if midlife neuroticism predicts dementia decades later, this would support a forward association—but also for informing how dementia prevention strategies could be tailored to specific age groups and psychological profiles. Many modifiable dementia risk factors, including depression and hypertension (both shown to mediate the neuroticism-dementia link [6]), vary in their association with dementia by age [11], with hypertension more strongly linked in midlife and depression associated with increased risk at both mid- and later life [12, 13]. Neuroticism’s influence may likewise vary across the life course. Although studies in older people (mean baseline age > 70) report positive associations [14–17], their short mean follow-up (<6 years) [14, 15, 17] raises concern for reverse causation.

Third, analyses of cognition may also offer insights into the directionality, as cognition is less affected by prodromal symptoms than diagnosis. Personality in adolescence predicts cognition 30 years later [18], and in mid- to later life, neuroticism has been consistently linked to poorer global cognition and memory [19]. However, domain-specific studies are scarce, often based on small sample sizes (*n* < 1000) [20–22] or brief assessments [6, 23]. Such analyses may strengthen evidence for dementia risk by demonstrating stronger associations with cognitive domains that are more sensitive to age-related decline, such as memory [24], and weaker associations with domains that remain relatively stable, like crystallised intelligence [25].

The European Prospective Investigation into Cancer in Norfolk (EPIC-Norfolk) cohort (>25 000 participants aged 39–79 at recruitment, 1993–97; followed to 2022) allows robust evaluation of time- and age-specific associations between neuroticism and incident dementia. We also examined interactions with genetic and lifestyle factors, mediation by health conditions and associations with domain-specific cognitive performance using a detailed battery.

## Methods

### Study population

The EPIC-Norfolk study is a population-based cohort of adults aged 39–79 years recruited between 1993 and 1997 [26]. Twenty-five thousand six hundred thirty-nine participants underwent an initial assessment [27], referred to as the first ‘health check’, which included self-administered written questionnaires on sociodemographic, lifestyle and medical history. Eighteen months later, participants were invited to complete the Health and Life Experiences Questionnaire (HLEQ), a postal questionnaire that included a measure of neuroticism; 20 921 responded between 1996 and 2000 (73.2% response rate from the eligible sample of 28 582) [28]. For our study, baseline was defined as the HLEQ completion date, with exclusions for invalid or missing dates (*n* = 9) and missing neuroticism data (*n* = 1237), leaving 19 678 dementia-free participants (see Appendix 1 for the study flow chart and Appendix 2 for the study timeline). Characteristics for participants with and without missing neuroticism data are provided in Appendix 3.

The EPIC-Norfolk study received ethical approval from the Norfolk Local Research Ethics Committee (05/Q0101/191) and the East Norfolk and Waveney NHS Research Governance Committee (2005EC07L). All participants provided signed informed consent.

### Neuroticism

Neuroticism was measured using the 12-item Eysenck Personality Questionnaire Revised-Short Form [29], with binary (‘yes’/‘no’) responses scored 1/0 to yield a total score of 0–12 (higher scores indicate greater neuroticism; distribution in Appendix 4).

### Dementia

Dementia was identified using national death records and Hospital Episode Statistics (HES) data available for the entire cohort, with diagnoses recorded using the International Classification of Diseases coding system (Appendix 5) [24]. For a small subset of participants, primary care and national mental healthcare records (including memory clinics) provided additional cases [30]. Dementia diagnoses by follow-up time and age of onset are shown in Appendix 6.

### Cognitive outcomes

The third EPIC-Norfolk health check (2006–11) included 8623 participants aged 48–92 years (~34% of the original cohort) [31]. Of these, 8585 completed at least one of eight cognitive tests (Appendix 7, test score distributions in Appendix 8). After excluding those with prevalent dementia (*n* = 4) or missing neuroticism (*n* = 1144), 7446 remained (Appendix 1).

Poor cognitive performance was defined as scoring below the 10th percentile on each test, except for the prospective memory test, where poor performance was defined as failing the task (a dichotomous outcome) [25]. A composite outcome grouped participants by the number of poor performances: 1, 2–3 or 4–8 tests, with none as the reference [24].

### Covariates and mediators

Socioeconomic covariates included the Townsend Deprivation Index (quintiles) and education (degree, A-level, O-level, <O-level). Lifestyle covariates included smoking status (never, former, current), alcohol consumption (never, former, ≤14 units per week and >14 units per week) and body mass index (BMI; normal [<25 kg/m^2^], overweight [≥25 and <30 kg/m^2^] and obese [≥30 kg/m^2^]); missing data were coded as separate categories.

Potential mediating conditions included depression, anxiety and stress-related disorders, hypertension and ischaemic heart disease (IHD), selected as modifiable dementia risk factors with evidence that neuroticism increases their risk but is not influenced by them [1–3]. Diabetes was selected as a negative control mediator, being a dementia risk factor not influenced by neuroticism and thus expected to explain little of the association. All conditions were based on self-reported doctor-diagnosed history at the first health check.

### Statistical analyses

Baseline characteristics were summarised by tertiles of neuroticism (0–2, 3–6, 7–12; Appendix 3), using mean and standard deviation (SD) for continuous variables or frequency and percentage for categorical variables.

Cox proportional hazards regression was used to examine the association between neuroticism scores and the risk of incident dementia, with age as the time scale and adjustments for sex and preselected socioeconomic and lifestyle covariates. Participants were followed from baseline until dementia, death or censoring date for HES/death records (31 March 2022). Proportional hazards were checked with scaled Schoenfeld residuals (Appendix 9). Nonlinearity was assessed using restricted cubic splines with knots at the 5th, 35th, 65th and 95th percentiles of the neuroticism score.

As no nonlinearity was detected, the neuroticism *z*-score (per SD) was used as the primary exposure. Models were first adjusted for age (as the time scale) and sex, then additionally for all covariates. Sensitivity analyses included: (i) using time since neuroticism assessment (i.e. follow-up time) as the time scale in Cox regression models; (ii) multiple imputation for missing exposure and covariate data; (iii) competing risk adjustment for nondementia death using the Fine–Gray model; and (iv) additional adjustment for extraversion. We stratified analyses by follow-up (<15, 15–19 and ≥ 20 years) and baseline age (<60, 60–69 and ≥ 70 years).

Analyses were also stratified by covariates and *APOE* ε4 status, testing multiplicative interactions with Wald tests. As subgroup analyses were based on preselected factors rather than data-driven testing, no correction for multiple comparisons was applied. We used a counterfactual mediation framework [32] to decompose the association between neuroticism and dementia into direct and indirect pathways and to estimate mediated proportions (details in Appendix 10).

For cognitive function, logistic regression assessed associations with each test, and multinomial logistic regression with composite outcomes, adjusting for age at the third health check, sex and the same covariates as dementia models. Neuroticism and covariates were based on the third health check data.

## Results

Among 19 678 participants (mean age 60.8, 56% women; Table 1), those with higher neuroticism were more likely to be younger, female, of lower socioeconomic status, consume less alcohol and smoke. Depression, anxiety and hypertension were also more common, while *APOE* ε4, ischaemic heart disease and diabetes showed little variation across tertiles.

**Table 1:** Baseline characteristics of participants

Characteristic	Overall	Neuroticism score tertile		
		1 (score 0–2)	2 (score 3–6)	3 (score 7–12)
*N*	19 678	6834	7548	5296
Age (mean [SD])	60.8 (9.3)	62.1 (9.1)	60.6 (9.2)	59.4 (9.2)
Women (%)	10 973 (55.8)	3049 (44.6)	4423 (58.6)	3501 (66.1)
Townsend Deprivation Index quintile (%)^a^				
1 (least deprived)	4052 (20.6)	1407 (20.6)	1576 (20.9)	1069 (20.2)
2	4021 (20.4)	1410 (20.6)	1546 (20.5)	1065 (20.1)
3	3953 (20.1)	1438 (21.0)	1480 (19.6)	1035 (19.5)
4	3911 (19.9)	1382 (20.2)	1511 (20.0)	1018 (19.2)
5 (Most deprived)	3667 (18.6)	1176 (17.2)	1406 (18.6)	1085 (20.5)
Missing	74 (0.4)	21 (0.3)	29 (0.4)	24 (0.5)
Education (%)				
Less than O-level^b^	7129 (36.2)	2337 (34.2)	2722 (36.1)	2070 (39.1)
O-level	2088 (10.6)	654 (9.6)	843 (11.2)	591 (11.2)
A-level^c^	7885 (40.1)	2876 (42.1)	2982 (39.5)	2027 (38.3)
Degree	2567 (13.0)	963 (14.1)	998 (13.2)	606 (11.4)
Missing	9 (<0.1)	4 (0.1)	3 (<0.1)	2 (<0.1)
Body mass index category (%)				
Normal	6804 (34.6)	2211 (32.4)	2672 (35.4)	1921 (36.3)
Overweight	7874 (40.0)	2935 (42.9)	2967 (39.3)	1972 (37.2)
Obese	2518 (12.8)	878 (12.8)	962 (12.7)	678 (12.8)
Missing	2482 (12.6)	810 (11.9)	947 (12.5)	725 (13.7)
Alcohol intake (%)				
Never	1005 (5.1)	367 (5.4)	373 (4.9)	265 (5.0)
Previous	1899 (9.7)	589 (8.6)	715 (9.5)	595 (11.2)
Current (≤ 14 units)	13 772 (70.0)	4733 (69.3)	5317 (70.4)	3722 (70.3)
Current (>14 units)	2819 (14.3)	1093 (16.0)	1073 (14.2)	653 (12.3)
Missing	183 (0.9)	52 (0.8)	70 (0.9)	61 (1.2)
Smoking (%)				
Never	9276 (47.1)	3226 (47.2)	3599 (47.7)	2451 (46.3)
Previous	8126 (41.3)	2889 (42.3)	3126 (41.4)	2111 (39.9)
Current	2126 (10.8)	665 (9.7)	768 (10.2)	693 (13.1)
Missing	150 (0.8)	54 (0.8)	55 (0.7)	41 (0.8)
* APOE* e4 carrier (%)	3795 (26.5)	1353 (26.5)	1449 (26.5)	993 (26.5)
Depression (%)	2705 (13.8)	323 (4.7)	837 (11.1)	1545 (29.2)
Anxiety and stress-related disorders (%)	582 (3.0)	79 (1.2)	182 (2.4)	321 (6.1)
Ischaemic heart disease (%)	1199 (6.1)	429 (6.3)	453 (6.0)	317 (6.0)
Hypertension (%)	2810 (14.3)	937 (13.7)	1090 (14.5)	783 (14.8)
Diabetes (%)	437 (2.2)	152 (2.2)	169 (2.2)	116 (2.2)

*N*, number of participants; SD, standard deviation.

^a^Townsend Deprivation Index: area-based measure (1991 census), calculated based on non-home/car ownership, unemployment and household overcrowding.

^b^Educational attainment at age 15.

^c^Educational attainment at age 17.

**Figure 1:**
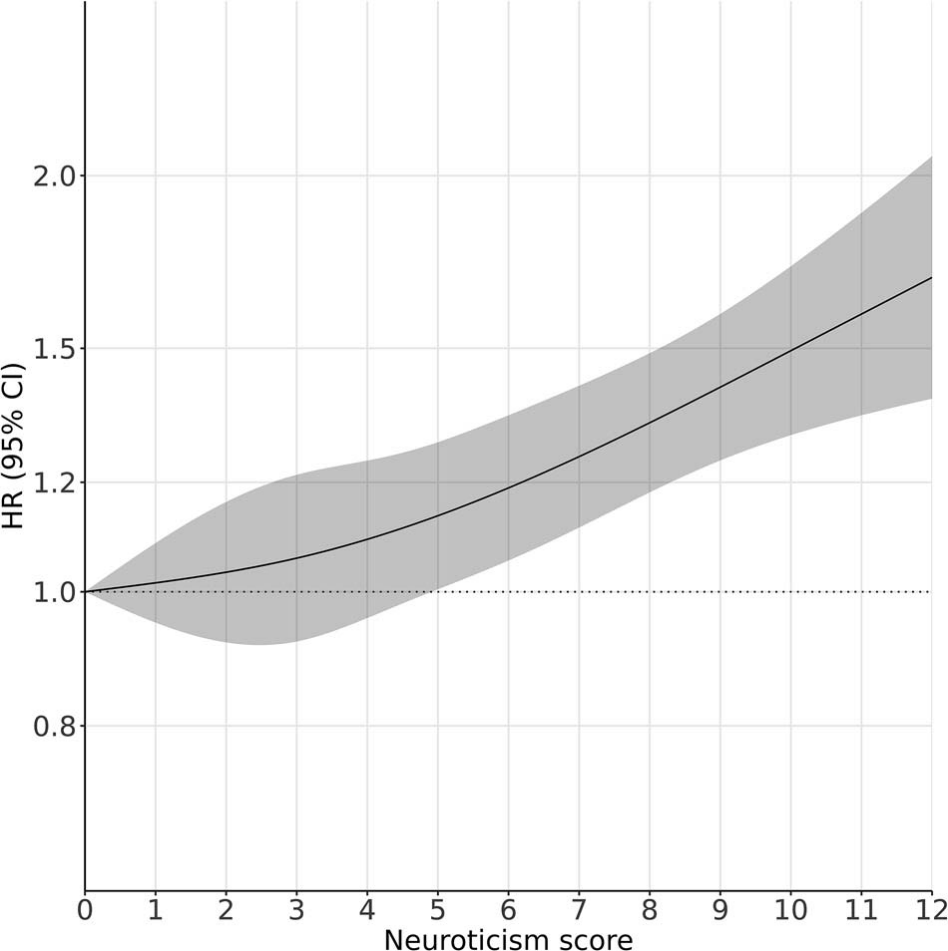
Association between neuroticism score and the risk for all-cause dementia. HR, hazard ratio; CI, confidence interval; estimates were adjusted for age (time scale), sex, education, quintiles of the Townsend Deprivation Index, smoking status, alcohol consumption and body mass index. Solid lines are adjusted HRs, with the area showing 95% CIs derived from restricted cubic spline regressions with four knots. The dashed line indicates a reference for no association at a hazard ratio of 1. The reference point for the neuroticism score is 0 (indicating the lowest level of neuroticism).

Over a median follow-up of 22.7 years, 2488 developed dementia, with risk increasing dose-responsively after covariate adjustment (Figure 1, *P* for nonlinearity .335). In the model adjusted for age and sex, a one-unit increase in neuroticism *z*-score was associated with a 15% higher risk of incident all-cause dementia (hazard ratio [HR] 1.15, 95% confidence interval [CI] [1.10–1.20]; Appendix 11) and remained similar after further adjustment for prespecified sociodemographic and lifestyle covariates (1.14 [1.10–1.19]). The association was consistent when using follow-up time as the time scale, after multiple imputation and with further adjustment for extraversion, and was slightly attenuated when accounting for the competing risk of death, consistent with the observed association between neuroticism and increased mortality (9139 deaths during follow-up; 1.04 [1.02–1.06]) (Appendix 12). Similar associations were found for Alzheimer’s disease (AD) (913 incident cases; 1.15, [1.08–1.23]) and vascular dementia (VaD) (643 incident cases; 1.16 [1.07–1.26]).

**Figure 2:**
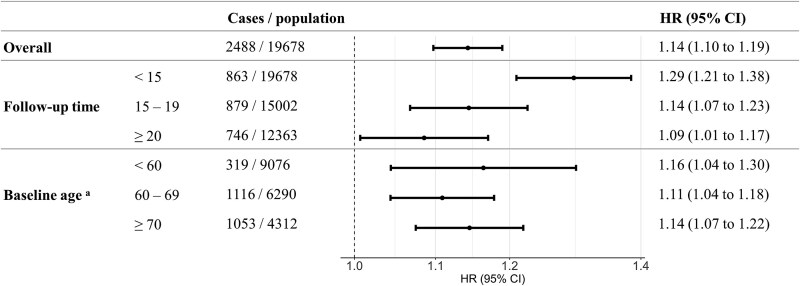
Association between neuroticism z-score and incident all-cause dementia stratified by follow-up period and baseline age. HR, hazard ratio; CI, confidence interval; estimates were adjusted for age (time scale), sex, education, quintiles of the Townsend Deprivation Index, smoking status, alcohol consumption and body mass index. ^a^There is no significant interaction between neuroticism and baseline age (*P* for interaction = .654). Age < 60 years: mean age 52.3 years (SD 4.3), mean follow-up 22.7 years (4.2). Age 60–69 years: mean age 64.5 years (2.9), mean follow-up 19.0 years (6.5). Age ≥ 70 years: mean age 73.5 years (2.5), mean follow-up 13.4 years (6.5).

The risk was highest in the first 15 years of follow-up (HR per SD 1.29 [1.21–1.38]; Figure 2) and remained modestly elevated after 20 years (1.09 [1.01–1.17]). Age-specific analyses showed similar increases across baseline age groups (<60: 1.16, [1.04–1.30]; 60–69: 1.11 [1.04–1.18]; ≥70: 1.14 [1.07–1.22]; *P* for interaction = .654).

**Figure 3:**
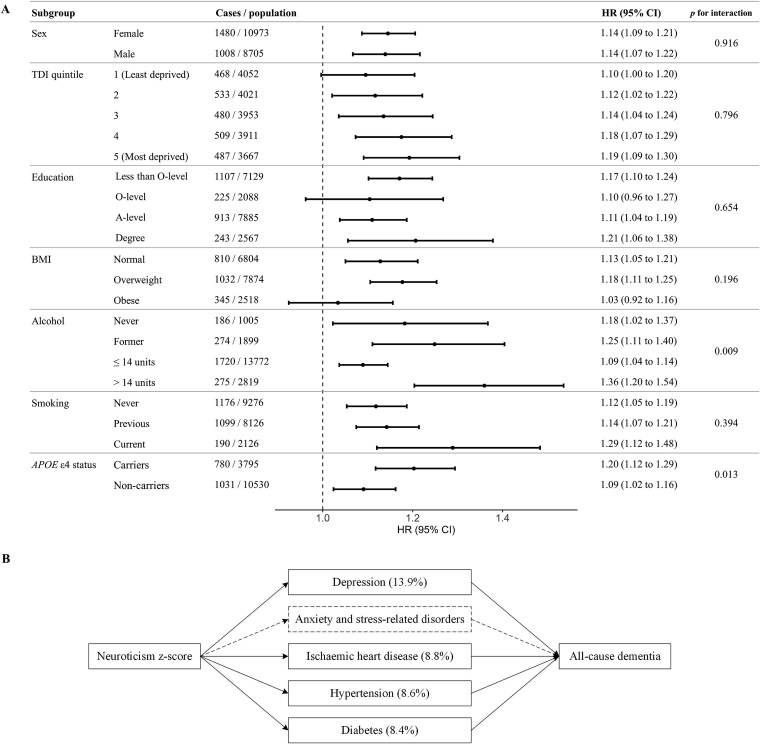
Stratified and mediation analyses of the association between neuroticism z-score and incident dementia. HR, hazard ratio; CI, confidence interval; TDI, Townsend deprivation index; BMI, body mass index. (a) Association between neuroticism *z*-score and all-cause dementia stratified by covariates and *APOE* ε4 status. (b) The association between neuroticism *z*-score and dementia mediated by a history of diseases at baseline. Analyses involving the *APOE* gene were restricted to participants of White ethnicity. Mediation proportions shown in parentheses. Mediators depicted with dashed borders and arrows indicate statistically insignificant mediation effects. See Appendix 13 for full mediation regression coefficients.

The association was consistent across sex, socioeconomic status, BMI and smoking status (Figure 3) but stronger in those consuming >14 units of alcohol per week (1.36 [1.20–1.54]) vs. ≤14: 1.09 [1.04–1.14], *P* for interaction = .009) and in former drinkers, though less pronounced. The association was also stronger among *APOE* ε4 carriers than noncarriers (1.20 [1.12–1.29]) vs. 1.09 [1.02–1.16], *P* for interaction = .013).

We detected a significant indirect (mediating) effect of selected conditions on the association between the neuroticism *z*-score and all-cause dementia (Figure 3; Appendix 13). The largest proportion was explained by depression (13.9%), followed by hypertension (8.8%), both of which were associated with increased dementia risk in this cohort and showed no substantial variation by age (Appendix 14); then IHD (8.6%), with diabetes contributing similarly (8.4%).

**Figure 4:**
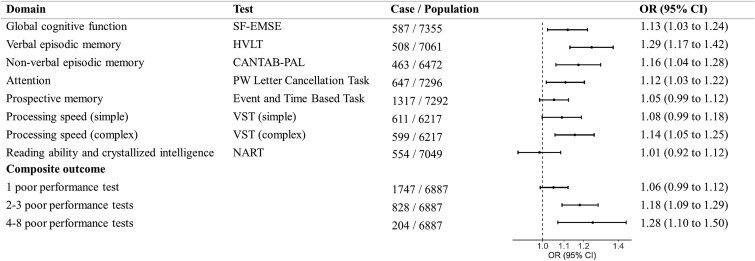
Association between neuroticism *z*-score and cognitive function. OR, odds ratio; CI, confidence interval; SF-EMSE, Short form-Extended Mental State Exam; HVLT, Hopkins Verbal Learning Test; CANTAB-PAL, Cambridge Neuropsychological Test Automated Battery Paired Associates Learning Test; VST, Visual Sensitivity Test; NART, National Adult Reading Test. Cross-sectional associations between the neuroticism *z*-score and cognitive performance were assessed using logistic regression, adjusting for age, sex, socioeconomic deprivation, education, smoking status, alcohol consumption and body mass index. ‘Case’ denotes the number of participants with poor performance (defined as scoring below the 10th percentile for each test, or task failure for prospective memory), and ‘Population’ denotes the number of participants with nonmissing scores for that test. Multinomial logistic regression was used to assess the association between neuroticism and the composite outcome. The composite outcome was defined as having 0, 1, 2–3 or 4–8 poor performance tests out of eight available measures. Participants with missing test data were retained if their completed tests allowed unambiguous classification (e.g. ≥4 poor tests); otherwise, the composite outcome was set to missing.

The 7446 participants included in the cognitive analyses (mean age 68.2, 55.2% female) had higher socioeconomic status and were less likely to be current alcohol consumers or smokers than baseline participants (Appendix 15). Characteristics by neuroticism level showed a similar distribution to baseline. Neuroticism at cognitive assessment correlated strongly with baseline levels (*r* = 0.73). Higher neuroticism was linked to poorer performance in multiple domains (Figure 4), most strongly in verbal (odds ratio [OR] 1.29, 95% confidence interval (CI) [1.17–1.42]) and nonverbal episodic memory (1.16 [1.04, 1.28]). Associations with prospective memory, processing speed and crystallised intelligence were nonsignificant. A dose–response association was observed for the composite outcome, with greater neuroticism linked to poorer performance across multiple domains.

## Discussion

In this population-based study of nearly 20 000 individuals aged 41–81 years followed for up to 26 years, higher neuroticism was associated with an increased risk of dementia, with each 1-SD increase in neuroticism score linked to a 14% higher risk. The association was stronger in the short term but remained significant even when the lag time between neuroticism measurement and dementia onset exceeded 20 years. A similarly increased risk was observed across age groups, including early midlife (41–60 years), late midlife (60–69 years) and later life (70–81 years). Among mid- to later-life adults without dementia, higher neuroticism was associated with poorer performance across multiple cognitive domains. Additionally, we found interactions between neuroticism and both *APOE* ε4 genotype and alcohol consumption, and potential mediation by mental and vascular conditions.

The association between neuroticism and increased dementia risk aligns with findings from a prior meta-analysis and two large UKB studies [4–6], and we further show that it remained significant when neuroticism was measured before age 60 or >20 years prior to diagnosis, supporting its role as a long-term risk factor rather than a psychological change emerging in the early stages of the disease. Two previous studies with >20 years’ follow-up reported significant associations, though with varying magnitudes. In one study (*N* = 800, baseline age 38–54, maximum follow-up 38 years), each 1-SD higher neuroticism was associated with a 4% increased risk of [33], likely underestimated due to survival bias. This is supported by prior evidence and by our finding that higher neuroticism is linked to earlier mortality before dementia onset [34, 35], with associations attenuated in Fine–Gray models. Another study (*N* = 1671, median baseline age 56.5, maximum follow-up 22 years) found a 37% increased risk of [36], though limited covariate adjustment may have inflated estimates. Additionally, a study of adolescents (mean age 15.8) found ‘calm’—an indicator of low neuroticism—was associated with a 5% lower risk of dementia after a mean follow-up of 69.5 years [37]. Importantly, a recent meta-analysis of prospective studies, less prone to recall bias, found no evidence that neuroticism increases during the long preclinical stage, when neuropathology is present but cognitive symptoms remain subclinical [10]. Together, these findings reinforce neuroticism as a potential risk factor for dementia and highlight the importance of early identification and intervention, as neuroticism typically stabilises in late adolescence [38].

In our study, the association between neuroticism and dementia risk was similar for those whose baseline in the study was in mid- or later life. Previous studies in older adults (baseline age > 70) reported significant associations between neuroticism and dementia risk [14–17], though nearly all had short follow-up durations (mean < 6 years). Our study extends this evidence by showing that the association remains robust in adults over 70, even with a substantially longer follow-up (mean 13.4 years). This suggests that neuroticism may help identify individuals at elevated risk of dementia even in later life and that addressing related factors such as depression and vascular health in these individuals could strengthen prevention efforts. While earlier exposure to high neuroticism could allow more time for cumulative damage (e.g. cerebrovascular injury), we did not observe a stronger association in younger age groups (e.g. baseline age < 60). This may reflect survival bias, where younger individuals with high neuroticism are less likely to survive to dementia onset [39].

The direct association between neuroticism and dementia is further supported by its link to poor cognitive function. In our analysis, we used a validated, detailed cognitive battery shown to predict future dementia [24]. A previous cross-sectional study (*N* = 2865) used a similarly detailed multi-domain cognitive battery (five domains) and reported comparable associations across domains [40]. However, its older population (mean age = 76) makes the findings more susceptible to reverse causation due to the inclusion of individuals in preclinical dementia stages. In contrast, participants in our analysis were a healthier, younger (median age = 68), dementia-free subgroup of EPIC-Norfolk, making our findings less prone to such bias. The association with poor global cognition and the observed dose–response relationship for the composite cognitive outcome suggest that neuroticism is linked to poor cognitive function across a broad range of domains, an important early indicator of cognitive decline [41]. The strongest association was found for verbal episodic memory, the strongest predictor of AD among all cognitive tests [24]; this is consistent with a large individual-participant meta-analysis (*n* = 120 640) showing that higher neuroticism is linked to faster decline in episodic memory [19]. In contrast, the lack of association with crystallised intelligence may reflect its relative stability with age compared to other cognitive domains [25].

Our mediation analyses aligned with a previous UKB study [6], showing that depression accounted for the largest proportion of the association, followed by vascular conditions, with diabetes contributing the least. The mediation proportions for depression (13.9%), IHD (8.6%) and hypertension (8.8%) were smaller than those reported previously in UKB (38.5%, 10.9% and 10.4%, respectively). These differences may reflect variation in cohort characteristics or the fact that these conditions show stronger associations with dementia in midlife [11], leading to larger mediation proportions in UKB, where participants were generally younger at baseline [6]. Given this, monitoring and improving mental and vascular health may offer a more direct and scalable approach to dementia prevention than modifying personality traits.

In our study, the association between neuroticism and dementia was particularly strong among *APOE* ε4 carriers and former or current heavy drinkers, consistent with both factors being associated with increased dementia risk [11]. This finding is consistent with a previous study (mean baseline age 78.6; 6.5 years follow-up) [42], in which most dementia cases occurred in the mid-80s, similar to our study and more representative of the general population [43]. In contrast, two studies (including one in UKB) with a younger baseline age (<65) and shorter follow-up (<15 years), where dementia onset occurred earlier (mid-70s), found no significant interaction [6, 36]. This may be because *APOE* ε4 is a strong determinant of earlier symptom onset [44], meaning dementia cases in the younger cohort had a stronger genetic component, potentially masking the additional contribution of neuroticism. As no correction for multiple comparisons was applied, interaction analyses should be interpreted as exploratory.

This study has several strengths, including a large population with a wide baseline age range and long follow-up, allowing us to examine whether the association between neuroticism and dementia remained stable across different age groups and time periods, helping to clarify the direction of the association. Additionally, the use of well-established cognitive tests for global and domain-specific function, which are sensitive to early cognitive changes preceding dementia symptoms, further strengthens our findings. However, this study has several limitations. First, healthier individuals with higher socioeconomic status were more likely to participate in EPIC-Norfolk baseline and follow-up examinations [45]. This may have led to underestimation of associations if individuals with higher neuroticism and poorer health were less likely to complete the postal questionnaire at 18 months or attend later waves for cognitive assessment. The low proportion of vascular conditions in this cohort also limits the validity of mediation analyses and generalisability to broader populations. Second, reliance on hospital inpatient and mortality records for dementia identification likely led to under-ascertainment, introducing misclassification bias that could bias effect estimates towards the null [46]. Third, while these records reliably capture all-cause dementia [47, 48], they perform poorly in distinguishing subtypes (e.g. AD or VaD) [47], limiting the validity of subtype-specific risk estimates. Fourth, as the HLEQ captured only neuroticism and extraversion, we were unable to account for the full range of personality traits. Fifth, potential mediators (disease history) were collected at the first health check, before neuroticism (18-month follow-up), so temporal ordering cannot be fully guaranteed. Sixth, as with all observational studies, causality cannot be inferred, and residual confounding is likely. Further research is needed in large, diverse cohorts with extended follow-up, longitudinal cognitive assessments and mediators measured after exposure to replicate these findings.

In conclusion, neuroticism was associated with an increased risk of dementia, remaining significant whether assessed in mid- or later life and even when assessed >20 years before dementia diagnosis. Neuroticism was also associated with poorer cognitive performance across multiple domains before dementia onset. Individuals with the *APOE* ε4 genotype or heavy alcohol consumption may be at particularly high risk, with depression and vascular conditions likely involved.

## Supplementary Material

aa-25-2365-File006_afaf339

## Data Availability

Upon publication, non-identifiable data can be made available to researchers on submission of a reasonable request to datasharing@mrc-epid.cam.ac.uk. Further details regarding the EPIC-Norfolk study data access policies can be found online: https://www.epic-norfolk.org.uk/for-researchers/data-sharing/data-access.

## References

[1] Howard DM, Adams MJ, Clarke TK et al. Genome-wide meta-analysis of depression identifies 102 independent variants and highlights the importance of the prefrontal brain regions. Nat Neurosci 2019;22:343–52. 10.1038/s41593-018-0326-7. 30718901 PMC6522363

[2] Zhang F, Baranova A, Zhou C et al. Causal influences of neuroticism on mental health and cardiovascular disease. Hum Genet 2021;140:1267–81. 10.1007/s00439-021-02288-x. 33973063

[3] Nagel M, Jansen PR, Stringer S et al. Meta-analysis of genome-wide association studies for neuroticism in 449,484 individuals identifies novel genetic loci and pathways. Nat Genet 2018;50:920–7. 10.1038/s41588-018-0151-7. 29942085

[4] Aschwanden D, Strickhouser JE, Luchetti M et al. Is personality associated with dementia risk? A meta-analytic investigation. Ageing Res Rev 2021;67:101269. 10.1016/j.arr.2021.101269. 33561581 PMC8005464

[5] Terracciano A, Aschwanden D, Passamonti L et al. Is neuroticism differentially associated with risk of Alzheimer's disease, vascular dementia, and frontotemporal dementia? J Psychiatr Res 2021;138:34–40. 10.1016/j.jpsychires.2021.03.039. 33819874 PMC8192471

[6] Gao Y, Amin N, van Duijn C et al. Association of neuroticism with incident dementia, neuroimaging outcomes, and cognitive function. Alzheimers Dement 2024;20:5578–89. 10.1002/alz.14071. 38984680 PMC11350007

[7] Beck ED, Yoneda T, James BD et al. Personality predictors of dementia diagnosis and neuropathological burden: An individual participant data meta-analysis. Alzheimers Dement 2024;20:1497–514. 10.1002/alz.13523. 38018701 PMC10947984

[8] Villemagne VL, Burnham S, Bourgeat P et al. Amyloid β deposition, neurodegeneration, and cognitive decline in sporadic Alzheimer's disease: a prospective cohort study. Lancet Neurol 2013;12:357–67. 10.1016/S1474-4422(13)70044-9. 23477989

[9] Robins Wahlin TB, Byrne GJ. Personality changes in Alzheimer's disease: a systematic review. Int J Geriatr Psychiatry 2011;26:1019–29. 10.1002/gps.2655. 21905097

[10] Terracciano A, Luchetti M, Karakose S et al. Meta-analyses of personality change from the preclinical to the clinical stages of dementia. Ageing Res Rev 2025;112:102852. 10.1016/j.arr.2025.102852. 40752776 PMC12358811

[11] Livingston G, Huntley J, Liu KY et al. Dementia prevention, intervention, and care: 2024 report of the <em>lancet</em> standing commission. Lancet 2024;404:572–628. 10.1016/S0140-6736(24)01296-0. 39096926

[12] Stafford J, Chung WT, Sommerlad A et al. Psychiatric disorders and risk of subsequent dementia: systematic review and meta-analysis of longitudinal studies. Int J Geriatr Psychiatry 2022;37:1–22. 10.1002/gps.5711. PMC9325434 35460299

[13] Fernández Fernández R, Martín JI, Antón MAM. Depression as a risk factor for dementia: a meta-analysis. J Neuropsychiatry Clin Neurosci 2023;36:101–9. 10.1176/appi.neuropsych.20230043. 38111332

[14] Wilson RS, Barnes LL, Bennett DA et al. Proneness to psychological distress and risk of Alzheimer disease in a biracial community. Neurology 2005;64:380–2. 10.1212/01.WNL.0000149525.53525.E7. 15668449

[15] Wilson RS, Arnold SE, Schneider JA et al. Chronic psychological distress and risk of Alzheimer's disease in old age. Neuroepidemiology 2006;27:143–53. 10.1159/000095761. 16974109

[16] Wilson RS, Schneider JA, Arnold SE et al. Conscientiousness and the incidence of Alzheimer disease and mild cognitive impairment. Arch Gen Psychiatry 2007;64:1204–12. 10.1001/archpsyc.64.10.1204. 17909133

[17] Duberstein PR, Chapman BP, Tindle HA et al. Personality and risk for Alzheimer's disease in adults 72 years of age and older: a 6-year follow-up. Psychol Aging 2011;26:351–62. 10.1037/a0021377. 20973606 PMC3115437

[18] Sutin AR, Stephan Y, Luchetti M et al. Self-reported and mother-rated personality traits at age 16 are associated with cognitive function measured concurrently and 30 years later. Psychol Med 2022;52:3854–64. 10.1017/S0033291721000672. PMC8435053 33706817

[19] Sutin AR, Brown J, Luchetti M et al. Five-factor model personality traits and the trajectory of episodic memory: individual-participant meta-analysis of 471,821 memory assessments from 120,640 participants. J Gerontol B Psychol Sci Soc Sci 2023;78:421–33. 10.1093/geronb/gbac154. 36179266 PMC9985335

[20] Wettstein M, Wahl HW, Siebert J et al. Still more to learn about late-life cognitive development: how personality and health predict 20-year cognitive trajectories. Psychol Aging 2019;34:714–28. 10.1037/pag0000374. 31259564

[21] Chapman BP, Benedict RH, Lin F et al. Personality and performance in specific neurocognitive domains among older persons. Am J Geriatr Psychiatry 2017;25:900–8. 10.1016/j.jagp.2017.03.006. 28456386 PMC5647872

[22] Caselli RJ, Dueck AC, Locke DEC et al. Impact of personality on cognitive aging: a prospective cohort study. J Int Neuropsychol Soc 2016;22:765–76. 10.1017/S1355617716000527. 27346168

[23] Desai P, Beck T, Krueger KR et al. Neuroticism, physical activity, and cognitive functioning in a population-based cohort of older adults. BMC Geriatr 2023;23:717. 10.1186/s12877-023-04399-8. 37926833 PMC10626783

[24] Hayat SA, Luben R, Khaw KT et al. The relationship between cognitive performance using tests assessing a range of cognitive domains and future dementia diagnosis in a British cohort: a ten-year prospective study. J Alzheimers Dis 2021;81:123–35. 10.3233/JAD-210030. 33867360 PMC8203214

[25] Hayat SA, Luben R, Dalzell N et al. Cross sectional associations between socio-demographic factors and cognitive performance in an older British population: the European investigation of cancer in Norfolk (EPIC-Norfolk) study. PLoS One 2016;11:e0166779. 10.1371/journal.pone.0166779. 27930656 PMC5145160

[26] Day N, Oakes S, Luben R et al. EPIC-Norfolk: study design and characteristics of the cohort. European prospective investigation of cancer. Br J Cancer 1999;80:95–103. 10466767

[27] Khaw K-T, Wareham N, Bingham S et al. Combined impact of health behaviours and mortality in men and women: the EPIC-Norfolk prospective population study. PLoS Med 2008;5:e12. 10.1371/journal.pmed.0050012. 18184033 PMC2174962

[28] Surtees PG, Wainwright NW, Khaw KT et al. Functional health status, chronic medical conditions and disorders of mood. Br J Psychiatry 2003;183:299–303. 10.1192/bjp.183.4.299. 14519607

[29] Eysenck SBG, Eysenck HJ, Barrett P. A revised version of the psychoticism scale. Personal Individ Differ 1985;6:21–9. 10.1016/0191-8869(85)90026-1.

[30] Hayat S, Luben R, Khaw KT et al. Evaluation of routinely collected records for dementia outcomes in UK: a prospective cohort study. BMJ Open 2022;12:e060931. 10.1136/bmjopen-2022-060931. PMC9204445 35705339

[31] Hayat SA, Luben R, Moore S et al. Cognitive function in a general population of men and women: a cross sectional study in the European investigation of cancer-Norfolk cohort (EPIC-Norfolk). BMC Geriatr 2014;14:142. 10.1186/1471-2318-14-142. 25527303 PMC4349767

[32] Lange T, Vansteelandt S, Bekaert M. A simple unified approach for estimating natural direct and indirect effects. Am J Epidemiol 2012;176:190–5. 10.1093/aje/kwr525. 22781427

[33] Johansson L, Guo X, Duberstein PR et al. Midlife personality and risk of Alzheimer disease and distress: a 38-year follow-up. Neurology 2014;83:1538–44. 10.1212/WNL.0000000000000907. 25274849

[34] Wilson RS, Krueger KR, Gu L et al. Neuroticism, extraversion, and mortality in a defined population of older persons. Psychosom Med 2005;67:841–5. 10.1097/01.psy.0000190615.20656.83. 16314587

[35] Shipley BA, Weiss A, Der G et al. Neuroticism, extraversion, and mortality in the UK health and lifestyle survey: a 21-year prospective cohort study. Psychosom Med 2007;69:923–31. 10.1097/PSY.0b013e31815abf83. 17991814

[36] Terracciano A, Sutin AR, An Y et al. Personality and risk of Alzheimer's disease: new data and meta-analysis. Alzheimers Dement 2014;10:179–86. 10.1016/j.jalz.2013.03.002. 23706517 PMC3783589

[37] Chapman BP, Huang A, Peters K et al. Association between high school personality phenotype and dementia 54 years later in results from a national US sample. JAMA Psychiatry 2020;77:148–54. 10.1001/jamapsychiatry.2019.3120. 31617877 PMC6802373

[38] Bleidorn W, Schwaba T, Zheng A et al. Personality stability and change: a meta-analysis of longitudinal studies. Psychol Bull 2022;148:588–619. 35834197 10.1037/bul0000365

[39] Graham EK, Rutsohn JP, Turiano NA et al. Personality predicts mortality risk: An integrative data analysis of 15 international longitudinal studies. J Res Pers 2017;70:174–86. 10.1016/j.jrp.2017.07.005. 29230075 PMC5722274

[40] Sutin AR, Stephan Y, Luchetti M et al. Five-factor model personality traits and cognitive function in five domains in older adulthood. BMC Geriatr 2019;19:343. 10.1186/s12877-019-1362-1. 31805866 PMC6896269

[41] Zaninotto P, Batty GD, Allerhand M et al. Cognitive function trajectories and their determinants in older people: 8 years of follow-up in the English longitudinal study of ageing. J Epidemiol Community Health 2018;72:685–94. 10.1136/jech-2017-210116. 29691286 PMC6204948

[42] Dar-Nimrod I, Chapman BP, Franks P et al. Personality factors moderate the associations between apolipoprotein genotype and cognitive function as well as late onset Alzheimer disease. Am J Geriatr Psychiatry 2012;20:1026–35. 10.1097/JGP.0b013e318267016b. 23079898 PMC4184145

[43] Mukadam N, Marston L, Lewis G et al. Incidence, age at diagnosis and survival with dementia across ethnic groups in England: a longitudinal study using electronic health records. Alzheimers Dement 2023;19:1300–7. 10.1002/alz.12774. 36047605

[44] Polsinelli AJ, Logan PE, Lane KA et al. APOE ε4 carrier status and sex differentiate rates of cognitive decline in early- and late-onset Alzheimer's disease. Alzheimers Dement 2023;19:1983–93. 10.1002/alz.12831. 36394443 PMC10182251

[45] Hayat SA, Luben R, Keevil VL et al. Cohort profile: a prospective cohort study of objective physical and cognitive capability and visual health in an ageing population of men and women in Norfolk (EPIC-Norfolk 3). Int J Epidemiol 2014;43:1063–72. 10.1093/ije/dyt086. 23771720 PMC4121549

[46] Copeland KT, Checkoway H, McMichael AJ et al. Bias due to misclassification in the estimation of relative risk. Am J Epidemiol 1977;105:488–95. 10.1093/oxfordjournals.aje.a112408. 871121

[47] Wilkinson T, Schnier C, Bush K et al. Identifying dementia outcomes in UK biobank: a validation study of primary care, hospital admissions and mortality data. Eur J Epidemiol 2019;34:557–65. 10.1007/s10654-019-00499-1. 30806901 PMC6497624

[48] Sommerlad A, Perera G, Singh-Manoux A et al. Accuracy of general hospital dementia diagnoses in England: sensitivity, specificity, and predictors of diagnostic accuracy 2008-2016. Alzheimers Dement 2018;14:933–43. 10.1016/j.jalz.2018.02.012. 29703698 PMC6057268

