# Supplementary material for: Association of neuroticism with incident dementia and cognitive function: 26-year follow-up of EPIC-Norfolk study
Source: Age Ageing. 2025 Nov 23;54(11):afaf339. doi: 10.1093/ageing/afaf339 (PMC12640544; doi:10.1093/ageing/afaf339)
Supplement: aa-25-2365-File006_afaf339 [file aa-25-2365-file006_afaf339.docx]

**Association of neuroticism with incident dementia and cognitive function: 26 year follow-up of EPIC-Norfolk study**

Contents

[Appendix 1. Flow diagram of analyses 2](#_Toc209050732)

[Appendix 2. Timeline of neuroticism, cognitive, and dementia assessments in EPIC-Norfolk 3](#_Toc209050733)

[Appendix 3. Comparison of baseline characteristics between participants with and without neuroticism score data 4](#_Toc209050734)

[Appendix 4. Distribution of neuroticism score 5](#_Toc209050735)

[Appendix 5. ICD10 codes used to define dementia in EPIC-Norfolk 6](#_Toc209050736)

[Appendix 6. Distribution of incident dementia diagnoses 7](#_Toc209050737)

[Appendix 7. Cognitive tests used in the EPIC-Norfolk 8](#_Toc209050738)

[Appendix 8. Distribution of cognitive test scores 10](#_Toc209050739)

[Appendix 9. Plot of scaled Schoenfeld residuals 11](#_Toc209050740)

[Appendix 10. Mediation analyses 12](#_Toc209050741)

[Appendix 11. Age- and sex-adjusted associations of neuroticism with dementia and mortality outcomes 13](#_Toc209050742)

[Appendix 12. Sensitivity analyses of the association between neuroticism z-score and incident dementia 14](#_Toc209050743)

[Appendix 13. Mediation analyses for the association between neuroticism z-score, baseline disease history, and incident dementia 15](#_Toc209050744)

[Appendix 14. Associations of baseline depression and hypertension with risk of incident dementia 16](#_Toc209050745)

[Appendix 15. Characteristics of participants included in the analysis of cognitive outcomes 17](#_Toc209050746)

## Appendix 1. Flow diagram of analyses


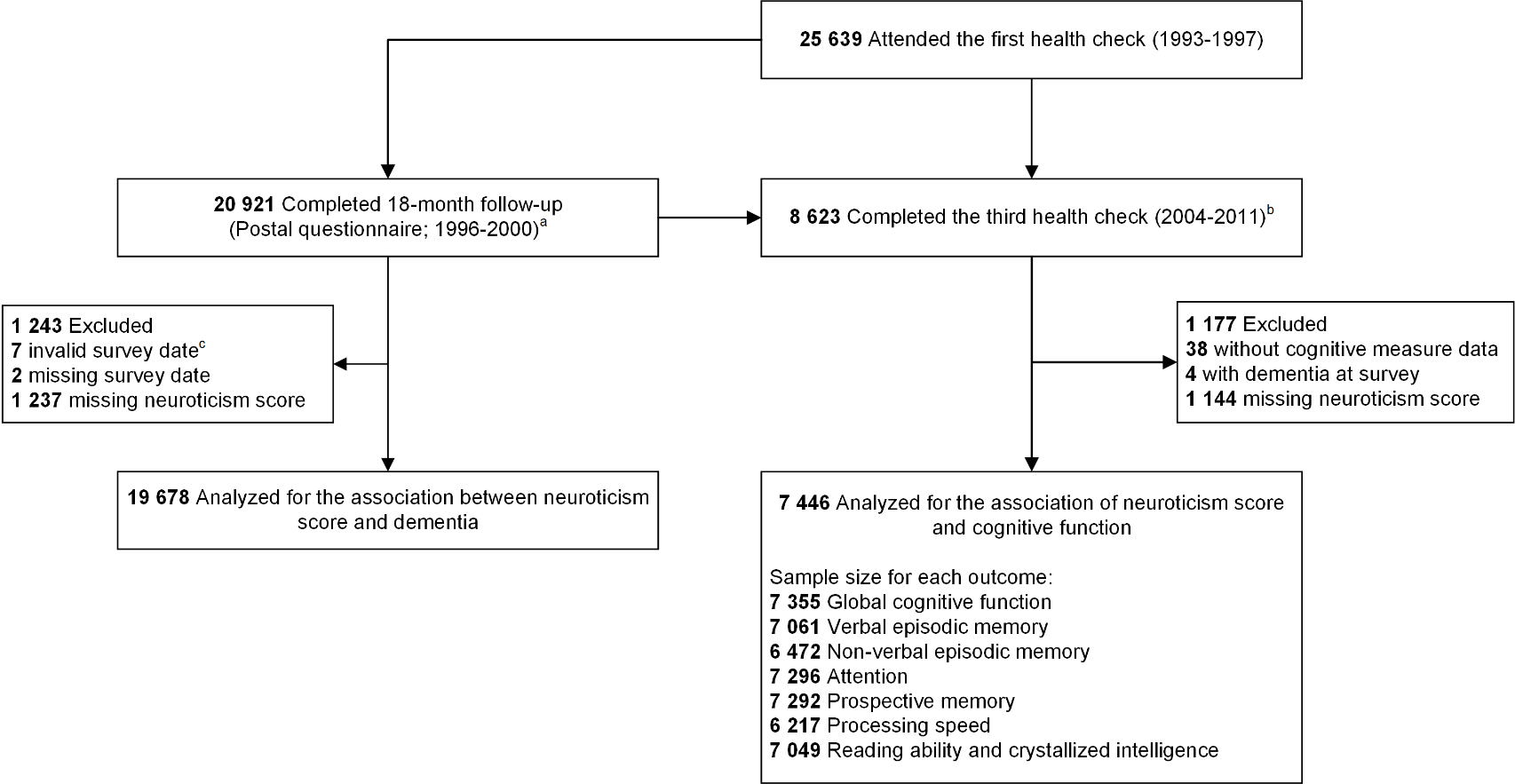


^a^ The 18-month follow-up recruitment occurred between 1994 and 1999. The Health and Life Experiences Questionnaire (HLEQ), which included the neuroticism assessment, was completed between 1996 and 2000.

^b^ The Third Health Check (3HC) included 8,623 participants from the original EPIC-Norfolk cohort, drawn from those who previously attended the baseline (1HC) and likely also completed the 18-month postal questionnaire.

^c^ Cases where the survey date is recorded as the same day as the date of death.

## Appendix 2. Timeline of neuroticism, cognitive, and dementia assessments in EPIC-Norfolk


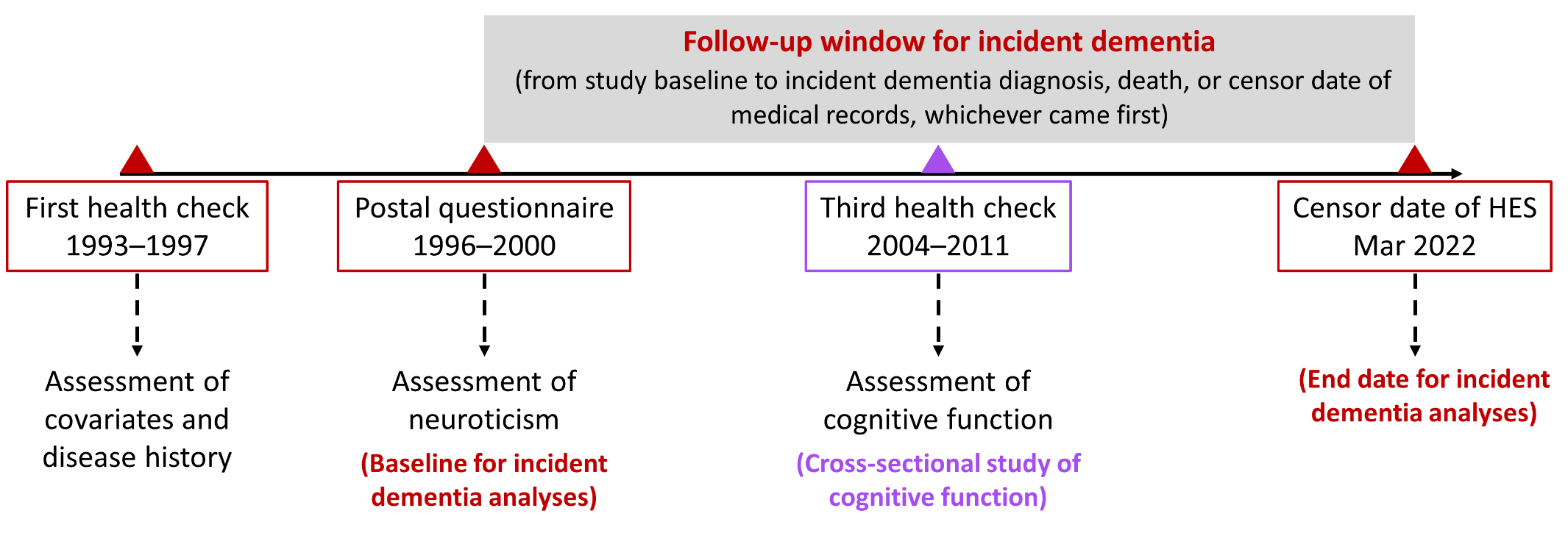


HES, Hospital Episode Statistics.

## Appendix 3. Comparison of baseline characteristics between participants with and without neuroticism score data

| Characteristic | Non-missing | Missing |
| --- | --- | --- |
| N | 19682 | 1237 |
| Age (mean [SD]) | 60.8 (9.3) | 64.2 (9.4) |
| Women (%) | 10977 (55.8) | 841 (68.0) |
| Townsend deprivation index quintile (%) ^a^ |  |  |
| 1 (Least deprived) | 4053 (20.6) | 218 (17.6) |
| 2 | 4021 (20.4) | 253 (20.5) |
| 3 | 3953 (20.1) | 226 (18.3) |
| 4 | 3911 (19.9) | 245 (19.8) |
| 5 (Most deprived) | 3670 (18.6) | 294 (23.8) |
| Missing | 74 (0.4) | 1 (0.1) |
| Education (%) |  |  |
| Less than O-level ^b^ | 7130 (36.2) | 560 (45.3) |
| O-level | 2088 (10.6) | 105 (8.5) |
| A-level ^c^ | 7887 (40.1) | 427 (34.5) |
| Degree | 2568 (13.0) | 145 (11.7) |
| Missing | 9 (0.0) | 0 (0.0) |
| Body mass index category (%) |  |  |
| Normal | 6807 (34.6) | 426 (34.4) |
| Overweight | 7874 (40.0) | 428 (34.6) |
| Obese | 2519 (12.8) | 166 (13.4) |
| Missing | 2482 (12.6) | 217 (17.5) |
| Alcohol intake (%) |  |  |
| Never | 1005 (5.1) | 105 (8.5) |
| Previous | 1901 (9.7) | 148 (12.0) |
| Current (≤ 14 units) | 13774 (70.0) | 820 (66.3) |
| Current (> 14 units) | 2819 (14.3) | 134 (10.8) |
| Missing | 183 (0.9) | 30 (2.4) |
| Smoking (%) |  |  |
| Never | 9278 (47.1) | 610 (49.3) |
| Previous | 8128 (41.3) | 482 (39.0) |
| Current | 2126 (10.8) | 126 (10.2) |
| Missing | 150 (0.8) | 19 (1.5) |
| *APOE* e4 Carrier (%) | 3796 (26.5) | 226 (27.8) |
| Depression (%) | 2706 (13.8) | 188 (15.2) |
| Anxiety and stress related disorders (%) | 582 (3.0) | 39 (3.2) |
| Ischaemic heart disease (%) | 1199 (6.1) | 88 (7.1) |
| Hypertension (%) | 2810 (14.3) | 215 (17.4) |
| Diabetes (%) | 437 (2.2) | 35 (2.8) |

N, number of participants; SD, standard deviation. ^a^ Townsend Deprivation Index: area-based measure (1991 census), calculated based on non-home/car ownership, unemployment, and household overcrowding; ^b^ Educational attainment at age 15; ^c^ Educational attainment at age 17.

## Appendix 4. Distribution of neuroticism score


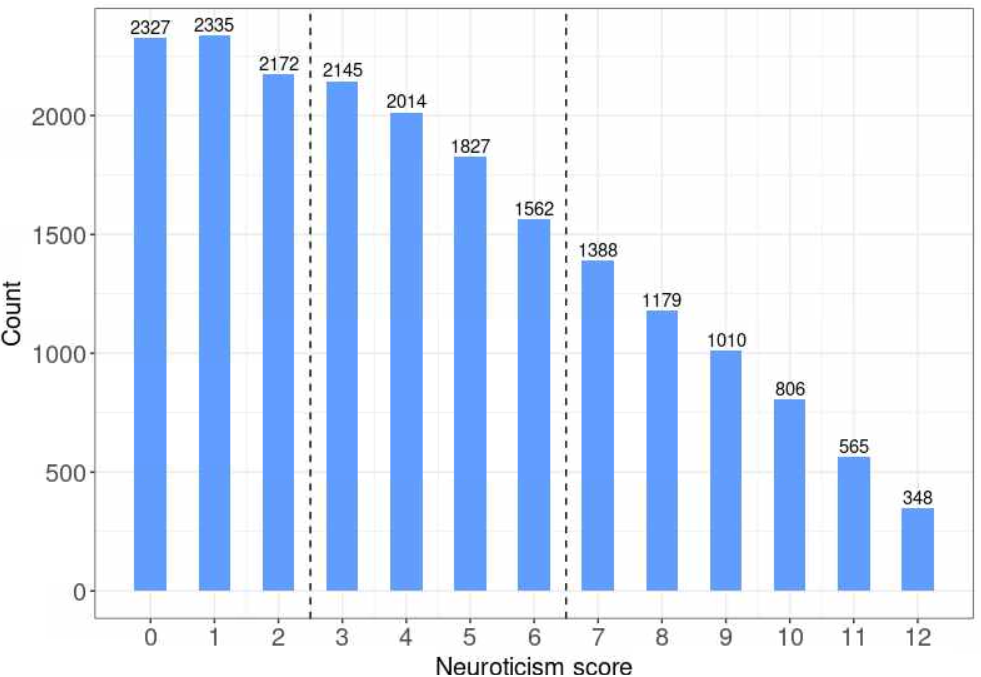


Bars show the number of participants (count) at each score level (range 0–12). Dashed lines divide the histogram into tertiles.

## Appendix 5. ICD10 codes used to define dementia in EPIC-Norfolk

| **ICD Code** | **ICD Description** |
| --- | --- |
| F00  G30 | Dementia in Alzheimer's disease  Alzheimer's disease |
| F00.0, G30.0 | Dementia in Alzheimer's disease with early onset |
| F00.1, G30.1 | Dementia in Alzheimer disease with late onset |
| F00.2 | Dementia in Alzheimer disease, atypical or mixed type |
| G30.8 | Other Alzheimer's disease |
| F00.9, G30.9 | Dementia in Alzheimer's disease, unspecified |
| F01 | Vascular dementia |
| F01.0 | Vascular dementia of acute onset |
| F01.1 | Multi-infarct dementia |
| F01.2 | Subcortical vascular dementia |
| F01.8 | Other vascular dementia |
| F01.9 | Vascular dementia, unspecified |
| F02 | Dementia in other diseases classified elsewhere |
| F02.0 | Dementia in Pick’s disease |
| F02.1 | Dementia in Creutzfeldt-Jakob disease |
| F02.2 | Dementia in Huntington's disease |
| F02.3 | Dementia in Parkinson's disease |
| F02.8 | Dementia in other specified diseases classified elsewhere |
| G31.0 | Frontotemporal dementia |
| G31.8 | Other specified degenerative diseases of nervous system. Grey-matter degeneration [Alpers]  Lewy body(ies)(dementia)(disease).Subacute necrotizing encephalopathy [Leigh] |
| F03 | Unspecified dementia |
| F05.1 | Delirium superimposed on dementia |
| F10.7 | Residual and late-onset psychotic disorder: includes Alcoholic dementia chronic alcoholic brain syndrome  Dementia and other milder forms of persisting impairment of cognitive functions |

ICD, International Classification of Diseases. Dementia diagnosis was provided as a derived variable by the EPIC-Norfolk team.

## Appendix 6. Distribution of incident dementia diagnoses


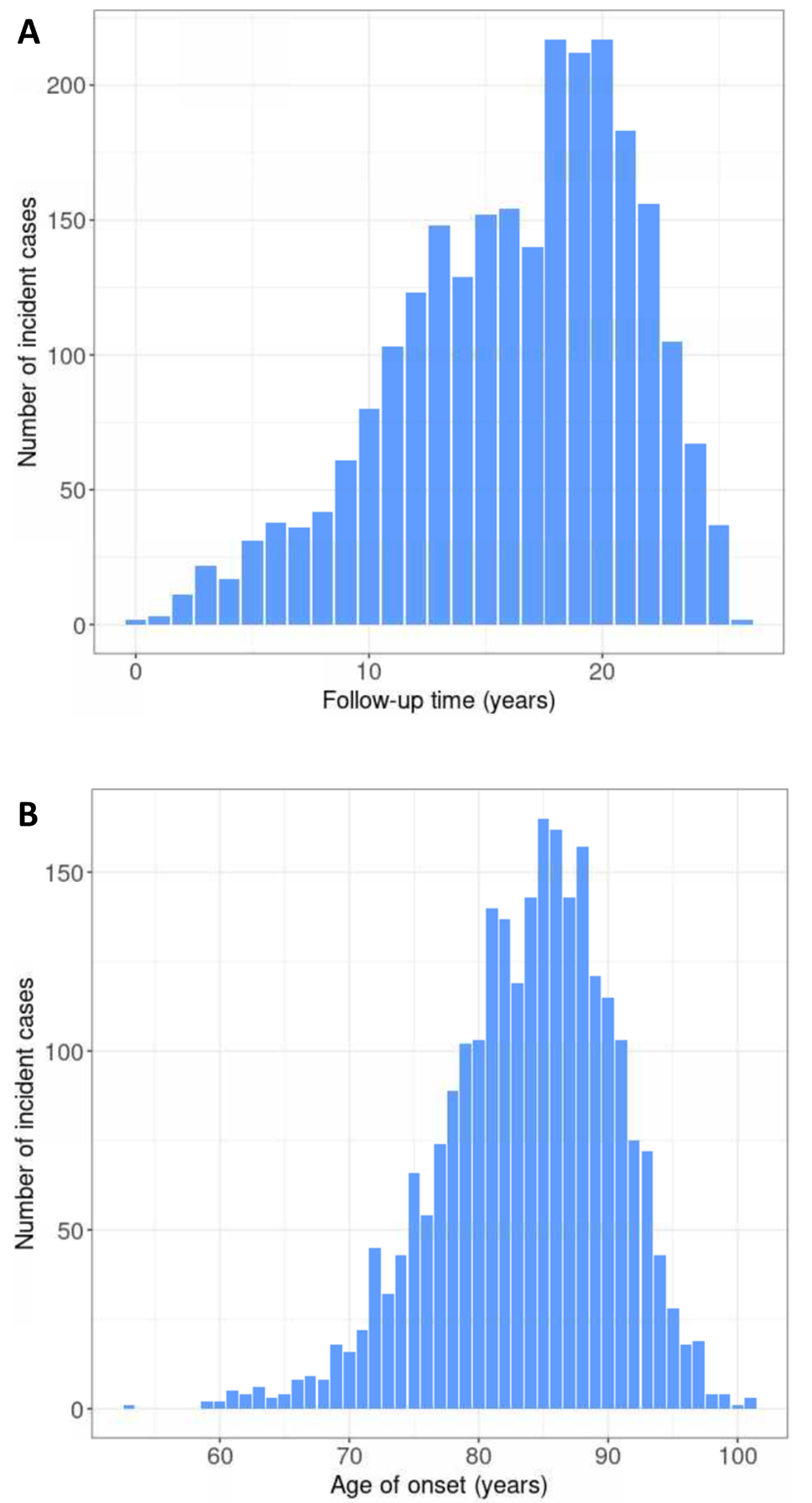


(A) Distribution of the number of incident cases by follow-up time since baseline. (B) Distribution of the number of incident cases by age of onset.

## Appendix 7. Cognitive tests used in the EPIC-Norfolk

| **Domain** | **Test** | **Outcome** |
| --- | --- | --- |
| Global cognitive function | A shortened version of the Extended Mental State Exam (SF-EMSE) ^1^ The SF-EMSE is a shorter version of the Extended Mental State Exam (EMSE), which itself builds upon the Mini Mental State Exam (MMSE) to better assess higher-functioning individuals. EPIC-Norfolk used 11 selected items to evaluate cognitive ability at the upper end of the spectrum, enabling derivation of a full MMSE score. | Test score (continuous) |
| Verbal episodic memory | Hopkins Verbal Learning Test (HVLT) ^2^ The HVLT assesses verbal recall and recognition using a 12-item word list from three semantic categories. Participants memorized the list, recalling it immediately after three separate presentations. Correct recalls were recorded for each trial and summed (maximum score: 36). | Score based on correctly recalled words (continuous) |
| Non-verbal episodic memory | Cambridge Neuropsychological Test Automated Battery Paired Associates Learning Test (CANTAB-PAL) ^3^ This task tests episodic memory and learning by requiring participants to recall the location of abstract patterns displayed sequentially on a touchscreen. Performance was evaluated across eight stages, with the primary outcome being the "first trial memory score" (FTMS), summing correct pattern-location matches on the first attempt. | First trial memory score (continuous) |
| Attention | PW Letter Cancellation Task (PW-Accuracy Score) ^4^ This test evaluates attention, processing speed, and accuracy. Participants scanned a grid of random letters (26 rows × 30 columns) to locate 72 target letters ("P" and "W") within one minute. Outcomes include speed (total letters searched) and accuracy (correct targets minus missed targets), with the latter used in this analysis. | Accuracy score (continuous) |
| Prospective memory | Event and Time Based Task^5^ This test assesses memory for future tasks, sensitive to early cognitive decline. Participants were instructed to seal and initial an envelope later in the session without prompting. Success was defined as completing at least one of the tasks correctly on their own. | Success (≥1 correct action without having to be prompted) or fail (dichotomous) |
| Processing speed | Visual Sensitivity Test (VST) - Simple and complex^6^ This test measures reaction times under two conditions: simple (detecting a triangle on a blank screen) and complex (detecting a triangle among moving dots). Reaction times were averaged across 70 trials, producing separate measures for simple (VST-Simple) and complex (VST-Complex) tasks. | Reaction time measured in milliseconds (continuous) ^a^ |
| Reading ability and crystallized intelligence | National Adult Reading Test (NART) ^7^ The NART estimates premorbid intelligence by assessing irregular word pronunciation, requiring prior vocabulary knowledge. Participants read 50 irregular words, with errors recorded (higher error scores indicate lower performance). EPIC-Norfolk used a short version, from which full scores were derived algorithmically. | NART Error Score (continuous) ^a^ |

^a^ Higher score corresponds to poorer performance.

1. Huppert FA, Cabelli ST, Matthews FE. Brief cognitive assessment in a UK population sample -- distributional properties and the relationship between the MMSE and an extended mental state examination. *BMC Geriatr*. May 4 2005;5:7. doi:10.1186/1471-2318-5-7

2. Brandt J. The hopkins verbal learning test: Development of a new memory test with six equivalent forms. *Clinical Neuropsychologist*. 1991/04/01 1991;5(2):125-142. doi:10.1080/13854049108403297

3. Fowler KS, Saling MM, Conway EL, Semple JM, Louis WJ. Computerized neuropsychological tests in the early detection of dementia: prospective findings. *J Int Neuropsychol Soc*. Mar 1997;3(2):139-46.

4. Richards M, Kuh D, Hardy R, Wadsworth M. Lifetime cognitive function and timing of the natural menopause. *Neurology*. Jul 22 1999;53(2):308-14. doi:10.1212/wnl.53.2.308

5. Huppert FA, Johnson T, Nickson J. High prevalence of prospective memory impairment in the elderly and in early-stage dementia: Findings from a population-based study. *Applied Cognitive Psychology*. 2000/01/01 2000;14(7):S63-S81. doi:https://doi.org/10.1002/acp.771

6. Hogervorst E, Bandelow S, Schmitt J, et al. Caffeine improves physical and cognitive performance during exhaustive exercise. *Med Sci Sports Exerc*. Oct 2008;40(10):1841-51. doi:10.1249/MSS.0b013e31817bb8b7

7. Beardsall L, Brayne C. Estimation of verbal intelligence in an elderly community: a prediction analysis using a shortened NART. *Br J Clin Psychol*. Feb 1990;29(1):83-90. [doi:10.1111/j.2044-8260.1990.tb00851.x](https://doi.org/10.1111/j.2044-8260.1990.tb00851.x)

## Appendix 8. Distribution of cognitive test scores


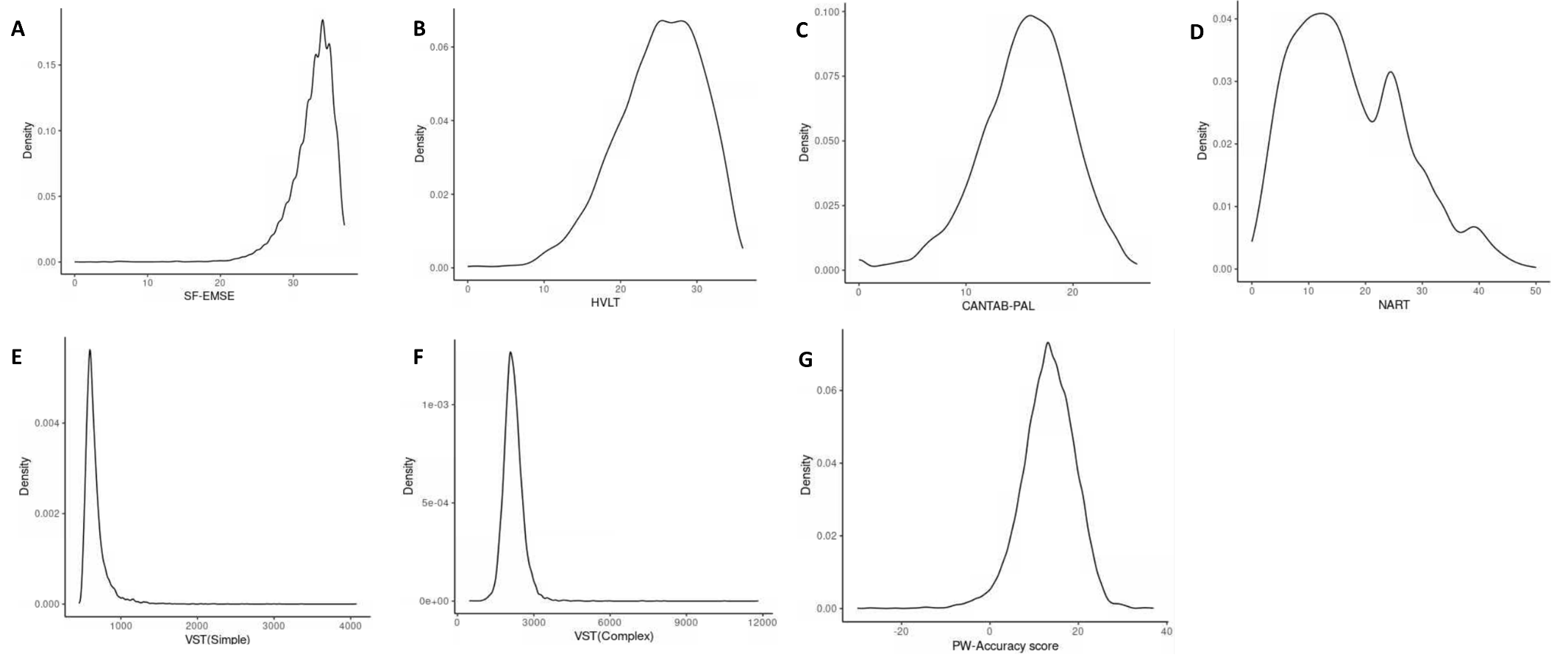


Density plots display the distribution of cognitive test scores (A–G), with the y-axis representing the relative probability (density) of observing a given score and the x-axis showing the score values. SF-EMSE, Short Form–Extended Mental State Exam; HVLT, Hopkins Verbal Learning Test; CANTAB-PAL, Cambridge Neuropsychological Test Automated Battery Paired Associates Learning Test; NART, National Adult Reading Test; VST, Visual Sensitivity Test; PW, Prospective Memory Accuracy Score.

## Appendix 9. Plot of scaled Schoenfeld residuals


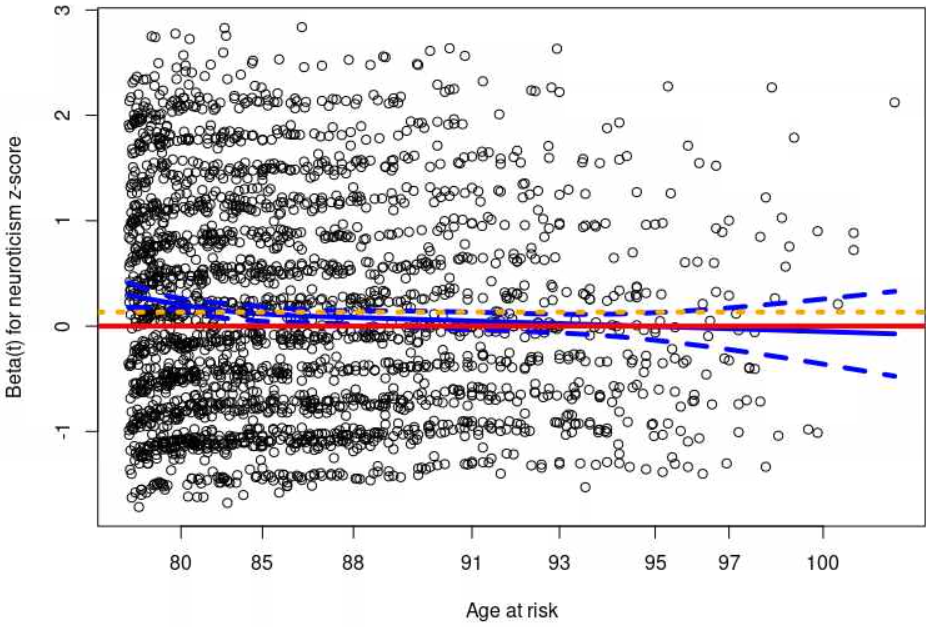


The points represent the scaled Schoenfeld residuals, which are the differences between an individual's neuroticism level at the time of an event and the corresponding risk-weighted average neuroticism level of all individuals at risk at that time, scaled inversely by their covariances. The blue smoothed curve represents the time-varying coefficient (β) for neuroticism, with dashed lines indicating the 95% confidence interval. The red line is a horizontal reference line at β = 0. The yellow dashed line indicates the average β over time, representing the point estimation from the Cox regression model.

## Appendix 10. Mediation analyses

We estimated the extent to which the association between neuroticism z-score (exposure, denoted as “A”) and incident dementia risk (outcome, denoted as “Y”) was mediated by the history of the following diseases (potential mediators, denoted as “M”): depression, anxiety and stress-related disorders, cardiovascular disease, hypertension, and diabetes. Each potential mediator was tested separately. We also adjusted for preselected covariates, including age, sex, education, Townsend deprivation index quintiles, smoking status, alcohol consumption, and body mass index (covariates, denoted as “C”).

This approach decomposes the total effect into natural direct effects and natural indirect effects within a counterfactual framework. Let Y_a,m_ denote the outcome value when A and M are set to A = a and M = m, respectively. The natural direct effect is defined as Y_a,M(a*)_ – Y_a*,M(a*)_, where a ≠ a*, i.e., the difference between the outcome value under exposure A = a and the outcome value if the same individual were instead exposed to A = a* with the mediator fixed at the level it would take under exposure level a*. The natural indirect effect is defined as Y_a,M(a)_ – Y_a,M(a*)_, reflecting the change in the outcome value when the exposure is fixed at level A = a, and the mediator is changed to its level under exposure A = a*. At the population level, the natural direct effect describes the effect of A on Y that is not due to changes in M, where M can vary among individuals according to all determinants except for A. Conversely, the natural indirect effect describes the effect of A on Y that is transmitted through M.

Marginal structural models (MSMs) are used to model the marginal expectation of a counterfactual outcome, which is implemented through the following steps:

1. Fitting logistic regression models to estimate the association between each potential mediator (history of a specific disease) and neuroticism z-score, conditioned on preselected covariates.
2. Constructing a new dataset by repeating each observation in the original data 5 times and replacing A* for subject *i* by randomly drawn exposures through resampling from the observed exposures.
3. Calculating the weight for each observation in the new dataset using the fitted models from step 1.

$$W_{i}^{C}=\frac{P\left( M=M_{i} | A=A_{i}^{*}, C=C_{i} \right)}{P\left( M=M_{i} | A=A_{i}, C=C_{i} \right)}$$

4. Fitting a weighted Cox model to estimate the hazard ratios (HR) for the direct and indirect effects, while controlling for preselected covariates. To account for the correlation between repeated observations, robust standard errors were calculated.
5. Calculating the proportion of the total effect that is explained by each individual mediator as HR_indirect effect_ / HR_total effect_.

## Appendix 11. Age- and sex-adjusted associations of neuroticism with dementia and mortality outcomes

| Outcome | Number of cases / 1000 person-years (IR) | | | Age- and sex-adjusted HR (95% CI) ^b^ |
| --- | --- | --- | --- | --- |
|  | **Tertile 1^a^ (score 0 – 2)** | **Tertile 2 (score 3 – 6)** | **Tertile 3 (score 7 – 12)** |  |
| Dementia | 875/130.2 (6.72) | 930/147.9 (6.29) | 683/105.1 (6.50) | 1.15 (1.10 to 1.20) |
| Alzheimer's disease | 299/131.4 (2.27) | 365/149.2 (2.45) | 249/106.3 (2.34) | 1.16 (1.08 to 1.23) |
| Vascular dementia | 238/131.7 (1.81) | 226/149.6 (1.51) | 179/106.5 (1.68) | 1.17 (1.08 to 1.27) |
| Death | 3456/132.0 (26.19) | 3432/150.0 (22.89) | 2251/106.8 (21.07) | 1.06 (1.04 to 1.08) |

IR, Incidence Rate; HR, hazard ratio; CI, confidence interval

^a^ Neuroticism score tertile

^b^ Association between neuroticism z-score and incident dementia and death, estimated using Cox regression models with age as the underlying time scale and adjusted for sex.

## Appendix 12. Sensitivity analyses of the association between neuroticism z-score and incident dementia

|  | HR (95% CI) |
| --- | --- |
| Follow-up time as time scale | 1.13 (1.09 to 1.18) |
| Multiple imputation | 1.14 (1.10 to 1.19) |
| Fine-Gray subdistribution model ^a^ | 1.08 (1.04 to 1.12) |
| Additional adjustment for extraversion ^b^ | 1.16 (1.11 to 1.21) |

HR, hazard ratio; CI, confidence interval

^a^ Fine-Gray subdistribution model was fit in R using the *crr* function in the *cmprsk* package. Neuroticism was associated with an increased risk of mortality (9,139 deaths during follow-up; HR 1.04, 95% CI [1.02 to 1.06]) after adjustment for the same pre-specified sociodemographic and lifestyle covariates.

^b^ Extraversion was assessed using the 12-item Eysenck Personality Questionnaire Revised-Short Form (EPQ-RS), included in the Health and Life Experiences Questionnaire (HLEQ) at the 18-month follow-up—the same time point as baseline neuroticism. Each item had a binary response ('yes' = 1, 'no' = 0), yielding a total score ranging from 0 to 12. We also examined the association between extraversion (z-score) and incident dementia. No significant associations were observed, either in models adjusted for age (as the time scale) and sex (HR 1.03, 95% CI [0.99 to 1.08]) or in fully adjusted models additionally accounting for pre-specified sociodemographic and lifestyle covariates (1.04, [0.99 to 1.08]).

## Appendix 13. Mediation analyses for the association between neuroticism z-score, baseline disease history, and incident dementia

| Mediator | Total effect (95% CI) | Direct effect (95% CI) | Indirect effect (95% CI) | Proportion mediated (%) |
| --- | --- | --- | --- | --- |
| Depression | 1.16 (1.11 to 1.21) | 1.13 (1.09 to 1.19) | 1.02 (1.00 to 1.04) | 13.9 |
| Anxiety | 1.15 (1.10 to 1.20) | 1.14 (1.09 to 1.19) | 1.01 (0.99 to 1.03) | - |
| Hypertension | 1.15 (1.10 to 1.20) | 1.14 (1.09 to 1.19) | 1.01 (1.00 to 1.03) | 8.8 |
| IHD | 1.15 (1.10 to 1.20) | 1.14 (1.09 to 1.19) | 1.01 (1.00 to 1.03) | 8.6 |
| Diabetes | 1.15 (1.10 to 1.20) | 1.14 (1.09 to 1.19) | 1.01 (1.00 to 1.03) | 8.4 |

“-” indicate insignificant mediating effect.

## Appendix 14. Associations of baseline depression and hypertension with risk of incident dementia

| Subgroups | Depression | | Hypertension | |
| --- | --- | --- | --- | --- |
|  | **HR (95% CI)** | ***p* for interaction** | **HR (95% CI)** | ***p* for interaction** |
| Overall | 1.22 (1.08 to 1.38) | - | 1.14 (1.03 to 1.26) | - |
| < 60 years | 1.35 (1.02 to 1.77) | 0.568 | 1.39 (1.00 to 1.95) | 0.605 |
| 60 – 69 years | 1.12 (0.93 to 1.34) |  | 1.19 (1.02 to 1.39) |  |
| ≥70 years | 1.21 (0.99 to 1.48) |  | 1.15 (1.00 to 1.33) |  |

Hazard ratios (HRs) and 95% confidence intervals (CIs) for the association of depression and hypertension with incident dementia, estimated using Cox proportional hazards models with age as the underlying time scale. Models were adjusted for sex and pre-specified socioeconomic and lifestyle covariates. Results are shown overall and stratified by baseline age (<60, 60–69, ≥70 years). p values for interaction were derived from Wald tests for multiplicative interaction terms.

## Appendix 15. Characteristics of participants included in the analysis of cognitive outcomes

| Characteristic | Overall | Neuroticism score tertile | | |
| --- | --- | --- | --- | --- |
|  |  | **1 (score 0 – 1)** | **2 (score 2 – 4)** | **3 (score 5 – 12)** |
| N | 7446 | 2531 | 2470 | 2445 |
| Age (mean [SD]) | 68.2 (8.1) | 69.4 (8.1) | 68.4 (8.0) | 66.7 (7.9) |
| Women (%) | 4112 (55.2) | 1095 (43.3) | 1416 (57.3) | 1601 (65.5) |
| Townsend deprivation index quintile (%) ^a^ |  |  |  |  |
| 1 (Least deprived) | 1860 (25.0) | 645 (25.5) | 628 (25.4) | 587 (24.0) |
| 2 | 1573 (21.1) | 561 (22.2) | 495 (20.0) | 517 (20.1) |
| 3 | 1478 (19.8) | 514 (20.3) | 488 (19.8) | 476 (19.5) |
| 4 | 1284 (17.2) | 388 (15.3) | 459 (18.6) | 437 (17.9) |
| 5 (Most deprived) | 1232 (16.5) | 417 (16.5) | 394 (16.0) | 421 (17.2) |
| Missing | 19 (0.3) | 6 (0.2) | 6 (0.2) | 7 (0.3) |
| Education (%) |  |  |  |  |
| Less than O-level ^b^ | 1866 (25.1) | 624 (24.7) | 589 (23.8) | 653 (26.7) |
| O-level | 902 (12.1) | 284 (11.2) | 298 (12.1) | 320 (13.1) |
| A-level ^c^ | 3328 (44.7) | 1136 (44.9) | 1117 (45.2) | 1075 (44.0) |
| Degree | 1349 (18.1) | 487 (19.2) | 465 (18.8) | 397 (16.2) |
| Missing | 1 (<0.1) | 0 (0) | 1 (<0.1) | 0 (0) |
| Body mass index category (%) |  |  |  |  |
| Normal | 2633 (35.4) | 830 (32.8) | 931 (37.7) | 872 (35.7) |
| Overweight | 2262 (30.4) | 878 (34.7) | 700 (28.3) | 684 (28.0) |
| Obese | 1419 (19.1) | 454 (17.9) | 461 (18.7) | 504 (20.6) |
| Missing | 1132 (15.2) | 369 (14.6) | 378 (15.3) | 385 (15.7) |
| Alcohol intake (%) |  |  |  |  |
| Never | 373 (5.0) | 133 (5.3) | 114 (4.6) | 126 (5.2) |
| Previous | 780 (10.5) | 264 (10.4) | 228 (9.2) | 288 (11.8) |
| Current (≤ 14 units) | 5199 (69.8) | 1739 (68.7) | 1767 (71.5) | 1693 (69.2) |
| Current (> 14 units) | 822 (11.0) | 298 (11.8) | 280 (11.3) | 244 (10.0) |
| Missing | 272 (3.7) | 97 (3.8) | 81 (3.3) | 94 (3.8) |
| Smoking (%) |  |  |  |  |
| Never | 3661 (49.2) | 1214 (48.0) | 1245 (50.4) | 1202 (49.2) |
| Previous | 3378 (45.4) | 1205 (47.6) | 1079 (43.7) | 1094 (44.7) |
| Current | 302 (4.1) | 79 (3.1) | 108 (4.4) | 115 (4.7) |
| Missing | 105 (1.4) | 33 (1.3) | 38 (1.5) | 34 (1.4) |

N, number of participants; SD, standard deviation. ^a^ Townsend Deprivation Index: area-based measure (1991 census), calculated based on non-home/car ownership, unemployment, and household overcrowding; ^b^ Educational attainment at age 15; ^c^ Educational attainment at age 17.
